# Mechanical Response and Analysis of Cracking Process in Hybrid TRM Composites with Flax Textile and Curauá Fibres

**DOI:** 10.3390/polym13050715

**Published:** 2021-02-26

**Authors:** Giuseppe Ferrara, Marco Pepe, Romildo Dias Toledo Filho, Enzo Martinelli

**Affiliations:** 1 Department of Civil Engineering, University of Salerno, Via Giovanni Paolo II n.132, 84084 Fisciano, SA, Italy; giferrara@unisa.it (G.F.); mapepe@unisa.it (M.P.); 2 TESIS srl, Via Giovanni Paolo II n.132, 84084 Fisciano, SA, Italy; 3 Civil Engineering Department, COPPE, Federal University of Rio de Janeiro, P.O. Box 68506, CEP 21941-972 Rio de Janeiro, Brazil; toledo@coc.ufrj.br

**Keywords:** Natural Textile-Reinforced Mortars, FRCM, plant fibres, flax, curauá, sustainable composite

## Abstract

In recent years, the use of plant fibres in Textile-Reinforced Mortar (TRM) composites emerged as a valuable solution to increase their sustainability. Several studies carried out to mechanically characterize the so-called Natural TRMs, although showing promising results, also emphasised some drawbacks due to a severe deformability of the system and to durability issues. This study aims at improving the mechanical behaviour of Natural TRMs including impregnated flax textile (Flax TRMs) by the addition of short curauá fibres within the matrix. Flax TRM specimens were tested in tension to assess the influence of the fibre-reinforced mortar on the composite response. The crack pattern developed during the test was investigated via Digital Image Correlation analysis and by means of an analytical simplified model proposed by the authors. The addition of curauá fibres resulted in a denser crack pattern and in a significant decrease of the mean crack width (around 20%). The overall tensile response of Flax TRMs including curauá fibres resulted closer to the ideal three-linear behaviour of strain-hardening TRM composites with respect to the conventional Flax TRMs by also presenting an increase of dissipated energy of around 45%. This study paves the way for further analysis aimed at enhancing the mechanical performance of Natural TRMs adopting sustainable improvement techniques.

## 1. Introduction

Composites with inorganic matrices, generally referred to as Textile-Reinforced Mortar (TRM) systems, are getting more and more common for strengthening masonry structures, with a remarkable increase in the last decade due to their efficiency in enhancing bearing capacity against both in-plane an out-of-plane actions [1,2]. Several studies have been developed with the aim to define qualification procedures for the assessment of TRM systems subjected to tensile forces [3] and their bond behaviour with masonry substrates [4]. Moreover, scientific committees have formulated characterisation procedures for the definition of the mechanical behaviour of TRMs [5,6]. Nowadays, the increasing awareness towards environmental issues pushes toward sustainable solutions for producing building materials and the use of recycled and renewable resources with lower environmental impact is increasingly required [7,8].

In this context, the use of plant fibres as reinforcement in cement-based composites has considerably increased, giving rise to a new class of sustainable building materials defined as biocomposites [9,10], characterised by an embodied energy equal to about one-fifth of the one required to produce conventional composite systems [11]. Due to attractive characteristics, such us renewability, low embodied energy, biodegradability and low cost, the use of green composites is expected to rise even faster in the next years [12]. Several studies were carried out to explore the possible use of plant fibre-based textiles as internal reinforcement in composites, introducing the so-called Natural Textile-Reinforced Mortar (NTRM) systems. 

To this purpose, the mechanical behaviour of NTRMs was experimentally assessed by considering several types of fibres, such as sisal [13,14], flax [15,16,17], jute [18,19], coir, hemp [20] and cotton. Applications were also carried out on a larger scale of analysis by the assessment of the efficiency of NTRMs in improving the capacity of masonry walls subjected to horizontal forces, both in-plane and out-of-plane [21,22,23,24]. Although emphasising the great potential in the use of such promising materials, these studies also highlighted some issues, mainly related with a significant deformability and degradation phenomena in the long term, hindering their use in real-world civil engineering applications [15,16,25]. 

A promising solution aiming at overcoming these issues is represented by textile treatments consisting in the impregnation by polymeric substances [26,27,28]. These types of treatment can enhance the stiffness of textiles and lead to a tensile response of the composite system characterised by lower strains and less significant load drops induced by crack formation. 

The present study aims at investigating the mechanical behaviour of improved NTRM systems including flax textiles, hereafter defined as Flax TRMs. Specifically, not only an impregnating treatment of the reinforcing textile is adopted, but also short curauá fibres are added as distributed reinforcement inside the mortar mixture and their influence on the tensile behaviour of Flax TRMs is investigated.

Flax fibres are among the most used cellulosic fibres. They are obtained from the stems of the *Linum usitatissimum* plant. Produced from the prehistoric period, they are considered as the oldest and among the strongest of the cellulosic fibres [11]. Flax fibres are widely adopted in furniture materials, textile bed sheets, linen, interior decoration accessories and in composite systems in several fields (aeronautic, naval, automotive, etc.) [29]. Canada is the largest flax producer, while France and Belgium, being the top exporter countries of flax fibres, are considered as the leading manufacturers of these materials [30]. Among the several plant fibres, flax was adopted to produce the sustainable composite object of study due to the promising mechanical properties. Moreover, with flax textiles being widely manufactured in Europe, where an increasing attention is devoted toward innovative green solutions also in the civil engineering field, their use complies with the requirement of adopting local sources to produce sustainable materials.

The use of short plant fibres has proven to be an efficient solution to increase flexural strength in cementitious composites [31,32,33]. However, their use in NTRM composites, proposed in the present study, is an innovative aspect. In line with studies form the literature, prior to being used, both flax textile and short curauá fibres are properly treated to maximise their efficiency: the former is impregnated with a polymeric substance [28], whereas the latter are subjected to wetting and drying cycles and, then, to immersion in an alkaline solution [34,35,36]. The results of tensile tests carried out on Flax TRMs with and without short curauá fibres reinforcement are discussed. The investigation is conducted via Digital Image Correlation analysis, proven to be an efficient technique for TRM tensile behaviour identification [37], and by proposing a simplified analytical model for the assessment of the crack pattern.

The paper includes a section comprising the characteristics of the materials adopted within the study, detailing the treatment procedures utilised. Then, the results of the experimental investigation carried out to assess tensile strength of two series of Flax TRMs, respectively with and without short curauá fibres, is presented. Finally, the results are discussed, and a detailed analysis of the crack pattern is reported.

## 2. Materials and Methods

### 2.1. Textile

Flax textile was adopted as reinforcement in the TRM composite system under consideration. It included equally spaced threads in both the warp and weft axis, which creates a bidirectional fabric with basically equal strength along the two directions. The thread consisted of several filaments, considered as the smallest element of the textile. Table 1 reports the main physical and mechanical properties of both flax filaments and threads, including their coefficient of variation (Co.V.) determined in previous related studies [25].

**Figure 1:**
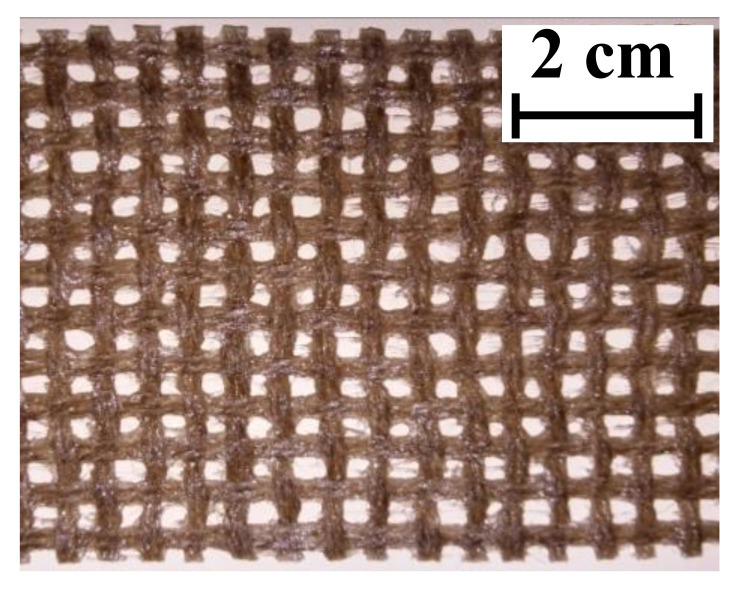
Impregnated flax textile.

Before being embedded in the mortar, the flax textile was coated with a polymeric substance. Specifically, a styrene butadiene polymer was adopted, which included a solid content of 49% ± 1% in weight, presented a viscosity of 50–350 mPas and a pH of 9 ± 0.5. Previous related studies showed the efficiency of this treatment in reducing the textile deformability, with consequent beneficial effects in the tensile response of the entire composite [27,28]. The coating treatment included the application of the polymeric substance, by means of a brush, on flax textile portions accurately stretched to make the impregnated textile straight. After the application of this coating compound, the textile was dried at a controlled temperature of 38 ± 2 °C in a ventilated chamber with an air flow velocity of 0.5 m/s for a period of 24 h. Flax strips adopted within the study were extracted from the impregnated textile after the treatment was completed. Figure 1 shows a sample of the impregnated flax textile adopted within the study.

Main geometric and mechanical properties of impregnated flax textile, assessed in previous related studies by tensile tests on 60 mm large strips [28], are listed in Table 1.

### 2.2. Short Fibres

Curauá short fibres were used to strengthen the mortar adopted as a matrix in the TRM composite system. This type of ligno-cellulosic fibre is obtained from the leaves of the *Ananás erectipholius* plant, an Amazonian plant found in northern Brazil and in the neighbouring South American countries [38]. The mortar was fibre-reinforced by plant fibres, instead of the most conventional steel or synthetic ones, in consistency with the principles of sustainability of the study. Among the several plant fibres, curauá was selected on the basis of literature studies showing that its application in mortar provides greater mechanical strength after matrix cracking [38]. Also, it was proven that composites with strain-hardening behaviour, reinforced by curauá fibres, are a promising solution in façade applications due to their toughness [39].

**Figure 2:**
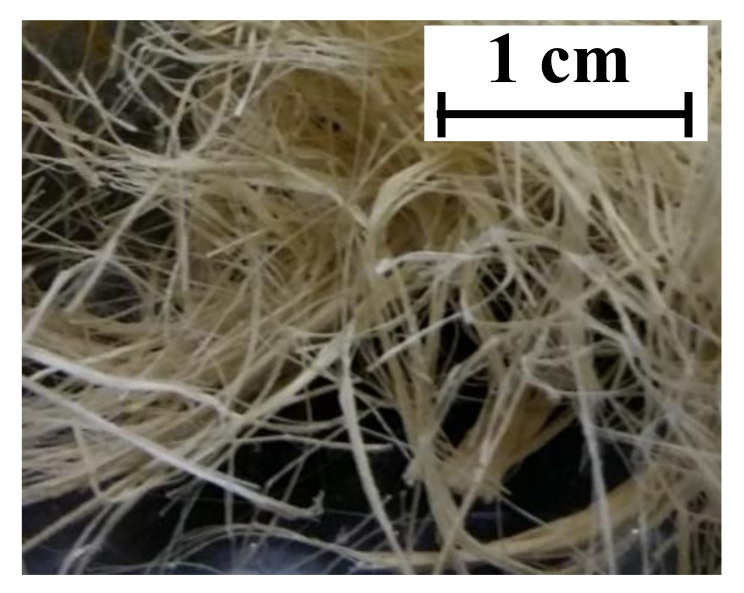
Short curauá fibres.

Prior to mixing fibres with the other mortar constituents, curauá fibres were subjected to physical and chemical treatments proven to be capable of improving their mechanical properties in the long term [35]. Firstly, three hornification cycles were applied to the filaments. Each cycle included 3 h of immersion in 80 °C water followed by 24 h of drying at 40 °C in a ventilated chamber. Secondly, fibres were subjected to a chemical treatment. It included the immersion of the filaments for 50 min in an alkaline solution with a concentration of 0.73% g/ml of Ca(OH)_2_, followed by 24 h of drying at 40 °C in a ventilated chamber. Figure 2 shows a group of curauá short fibres after the treatment. Main physical and mechanical properties of treated curauá filaments, obtained from Reference [36], are listed in Table 1.

### 2.3. Mortar

The matrix of the composite under investigation was a hydraulic lime mortar. The efficiency of the use of this type of mortar in TRM composites for the strengthening of masonry structural elements was highlighted by different studies in Reference [1]. Two different mortars were adopted in the study: plain mortar and short fibre-reinforced mortar. The plain mortar included a pre-mixed mixture of pure natural hydraulic lime as binder and fine aggregates with maximum diameter of 1.19 mm. The grain size distribution was defined to not undermine the proper penetration of the fresh mortar within the clear opening spaces of the flax textile adopted during the specimens’ casting. The amount of water of the mortar mix design was 19% of the premixed powder (including binder and aggregates), corresponding to a water-to-binder ratio of 0.6.

The fibre-reinforced mortar was obtained by including short curauá filaments during the mixture process. An amount of fibres equal to the 1% in weight of the fresh mortar was added. This quantity was considered as a reasonable compromise between a minimum amount to have a beneficial effect on the hardened mortar mechanical properties [38,40] and a maximum amount not preventing a proper workability at the fresh state. Mechanical properties at the fresh and hardened state for both the mortars adopted were assessed and listed in Table 1. Fresh state workability of the mortar was expressed in terms of spreading diameter obtained by flow table test after jolting the table 15 times [41]. Flexural and compression tests were carried out after 28 days from the casting to assess hardened mortar mechanical properties [42]. As expected, the addition of short fibres to the mix design resulted in a lower workability at the fresh state and in higher flexural strength at the hardened state.

### 2.4. TRM Composite System

Flax Textile-Reinforced Mortar (Flax TRM) with and without short fibres (SF) composite samples were created with the aim of assessing the tensile capacity of the material. Two series of specimens, differing in the type of matrix, were considered:

- Flax TRM: composite specimens including impregnated flax textile and plain mortar.
- SF_Flax TRM: composite specimens including impregnated flax textile and short curauá fibre-reinforced mortar.

Specimens had width of 60 mm and length of 500 mm and included two layers of impregnated flax textile strips having the same dimensions. Specimens were manufactured in properly designed moulders. 

The first layer of mortar was placed at a depth of approximately 2 mm. The textile was arranged onto the mortar by pressing with a roller to guarantee a proper impregnation of the grid in the fresh mortar. An intermediate layer of mortar, having a thickness of approximately 2 mm, was placed on the textile and, with the same procedure of the first one, the second layer of textile was arranged on it. Finally, the outer layer of mortar, of the same thickness as the others, was placed and the external surface was properly smoothed. 

**Figure 3:**
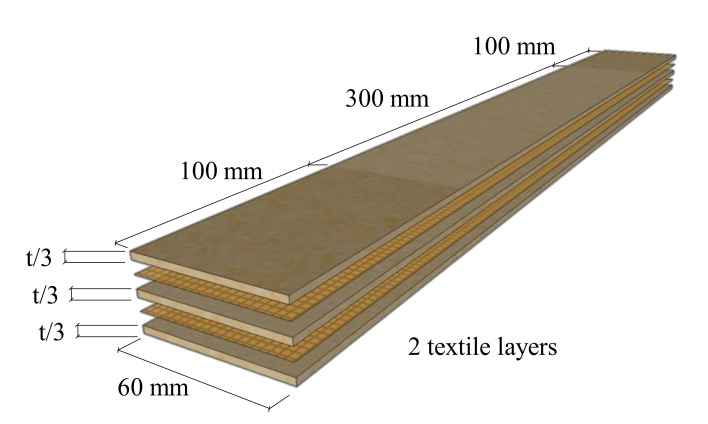
Geometry of Flax Textile-Reinforced Mortar (TRM) specimens.

SF_Flax TRM specimens resulted having a larger thickness due to the lower workability that hindered the manufacturing process. The mean thickness of the specimen, t, was of 8.1 mm (Co.V. 11%) for Flax TRM series, and 9.4 mm (Co.V. 6%) for SF_Flax TRM specimens, with a consequent fibre-reinforced ratio (ratio between textile and composite cross sections) respectively equal to 2.5% and 2.2%. Each series included 5 samples. Specimens had a gauge length of 300 mm and were anchored at each edge for a length of 100 mm. Figure 3 shows the geometry of Flax TRM specimens.

The tensile test set-up was arranged to reproduce the clevis configuration according to Rilem’s recommendations [5]. Two aluminium plates were glued by means of an epoxy resin to each edge of the specimen. A system comprising two bolts and a steel device reproduced two hinges, allowing rotation of the specimen in its plane and in the plane transversal to its axis. Thus, specimens were subjected to pure tensile load conditions, and no transversal compression was applied in the gripping area.

**Figure 4:**
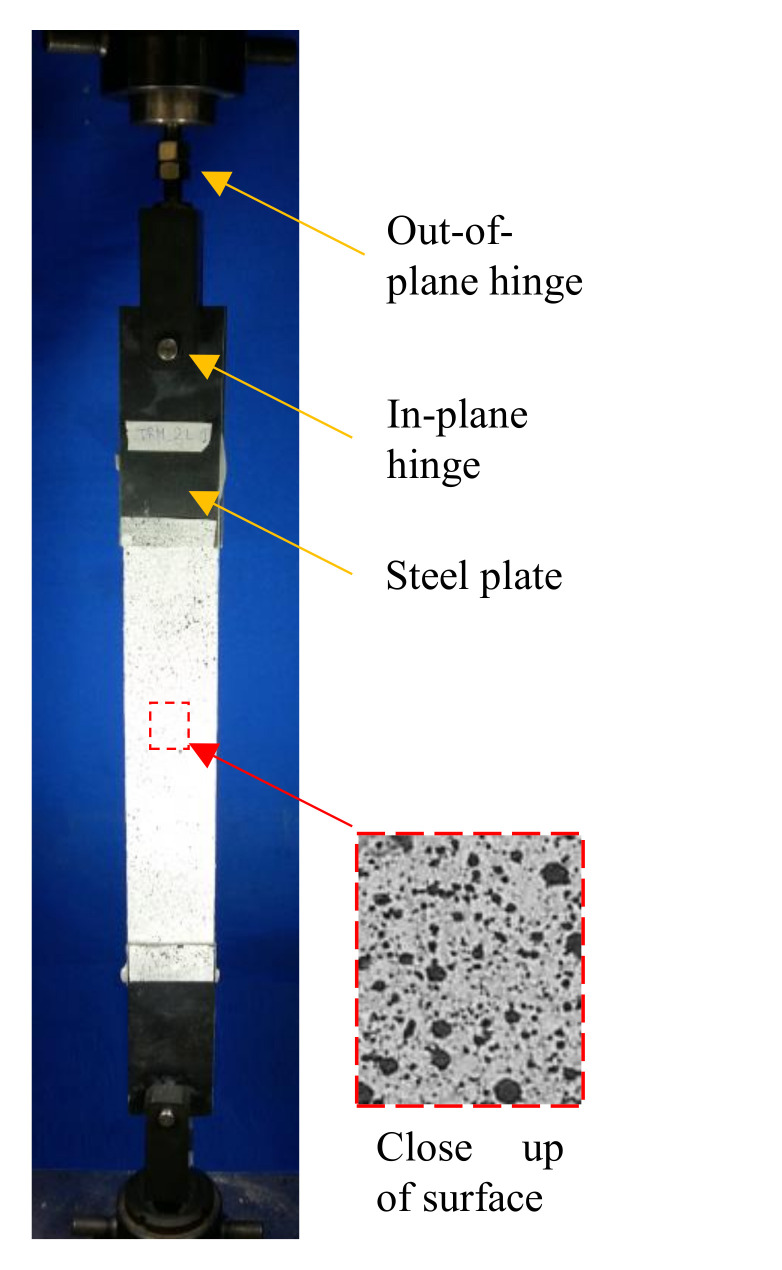
Flax TRM tensile test set-up.

The external surface of the specimens was treated by creating a white background and by painting a pattern of black dots randomly distributed in order to have pictures for Digital Image Correlation (DIC) analysis. The camera was placed at a focal distance from the specimen equal to 500 mm. Photos, having a resolution of 4310 × 2868 pixels (0.08 mm/pix), were taken with a rate of 3.75 photos/min. Tensile tests were carried out by means of a Schimadzu (model AG-Xplus, Shimadzu Corporation, Kyoto, Japan) universal testing machine with a maximum capacity of 10 kN, in displacement control with a rate of 0.3 mm/min. Figure 4 shows the test set-up adopted for the assessment of the tensile behaviour of the Flax TRM composites object of study.

## 3. Results and Discussion

### 3.1. Tensile Stress-Strain Response

**Figure 5:**
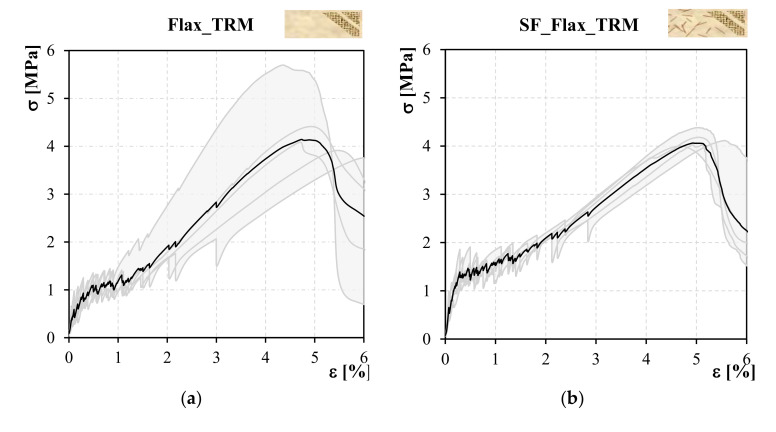
(**a**) Stress-strain curves concerning Flax_TRM specimens. (**b**) Stress-strain curves concerning SF_Flax_TRM specimens.

Tensile response of Flax TRM composites was expressed in terms of normal stresses–axial strain curves. Stresses were defined with respect to the entire transversal cross-section of the composite. Figure 5a,b shows stress-strain curves concerning the two series of specimens. For each series, the mean curve was plotted and the region comprising all the specimen curves was represented as well.

As expected, a tri-phase behaviour was observed for both series. Notably, the elastic phase, herein defined as stage I, showed a quasi-linear behaviour until the occurrence of the first crack within the mortar matrix. Subsequently, the phase characterised by the crack development, defined as stage II, presented several peaks. Each peak represented the onset of a crack, in correspondence with which a drop load occurred.

Once the entire crack pattern had developed, tensile response was mainly governed by the reinforcing textile entailing a strain-hardening behaviour up to failure (stage III). The points bounding the three stages were chosen as parameters describing the mechanical behaviour: the couple (*ε_1_*, *σ_1_*) represented the transition point between stages I and II, the couple (*ε_2_*, *σ_2_*) represented the transition point between stages II and III and the couple (*ε_max_*, *σ_max_*) represented the point corresponding to the maximum load (considered as the failure point).

All the specimens exhibited a failure mode including the development of several cracks, oriented along the transversal section, and crossing it for the entire thickness. The failure involved both slipping phenomena of the textile within the matrix in proximity of the gripping area, and tensile rupture of flax threads in proximity of a random cracked section. Stress-strain curves for both series of specimens confirm that the tensile response of Flax TRMs including impregnated textile exhibit lower deformability, notably in stage II, a wider crack pattern and less significant drop of the load corresponding to the crack opening, with respect to related studies in which the textile was adopted without treatment [15,28].

Tensile response of some specimens, especially with respect to stage III, significantly deviate from the mean stress-strain curve. This aspect is due to the type of impregnation treatment executed on the flax textile. The treatment was hand-made and resulted in a non-uniform coating application to all the impregnated textile strips. In this sense, the textile coating treatment needs to be somehow mechanised to guarantee a proper impregnation rate, and lower variability in the composite mechanical properties.

SF_Flax TRM specimens exhibited a mean stress value corresponding to the occurrence of the first crack, *σ_1_*, higher with respect to the Flax TRM specimens. As shown by the mean values in Table 2, the addition of short curauá fibres within the mortar matrix leads to an increase in stress *σ_1_* of about 10%. With the flexural strength of the short fibre-reinforced mortar being significantly higher than that of plain mortar, it was expected the first crack would occur at higher values of the stress within the mortar. The value of the normal stress within the SF_Flax TRM composites remains on average higher during the entire stage II with respect to Flax TRM specimens. 

This aspect of the mechanical response can be quantified by determining the energy *Γ_II_*, defined as the area underlying the force-displacement curve in stage II; in fact, it represents the energy dissipated during the development of the crack pattern. Mean values of *Γ_II_* for both series of specimens are shown in Table 2. SF_Flax TRM specimens exhibit a mean value higher by about 45% with respect to Flax TRM specimens. These aspects emphasised the higher capacity of the improved system to exhibit deformations without losing load capacity, a fundamental aspect especially in view of application of the composite on masonry elements to enhance their capacity to resist seismic actions. In fact, for this purpose, the dissipative capacity of the structural element needs to be improved. 

Regarding the strains, the two series of specimens do not present significant differences. In fact, the deformability of the composite is mainly governed by the textile (and the mortar stiffness), rather than by the short fibres added within the matrix.

Similarly, the stiffness in stage III, assuming mean values of 4.0 GPa (Co.V. 33%) and 3.8 GPa (Co.V. 6%) respectively, for Flax TRM and SF_Flax TRM specimens, is not significantly affected by the curauá short fibre addition. This confirms, as expected, that the response of the composite in stage III, once the crack pattern is completely developed, is mainly governed by the reinforcing textile rather than by the matrix. Both the systems, in line with conventional TRMs, presented a hardening behaviour during this phase. Once the composite is applied as a reinforcement system of structural elements, this hardening behaviour may contribute to increase the capacity of the strengthened members.

Textile exploitation ratio, defined as the ratio between the maximum stress in the textile during the test and its tensile strength, is slightly higher in SF_Flax TRM specimens, being 72% compared to the mean value of 65% shown by Flax TRM specimens. This higher exploitation of the textile may be due to the fact that the failure mode also involves slipping phenomena of the textile within the matrix. In this sense, the short fibre reinforcement may have improved this aspect, promoting a more uniform distribution of the stress through the composite cross-section, hence delaying damage phenomena of the matrix in proximity of the textile.

### 3.2. Analysis of the Crack Pattern

The parameters considered for the analysis of the crack pattern of TRM composites developed during the tensile test are the number of fissures along the free length of the specimens (also expressed as number of cracks for unit length) and their longitudinal width. Both parameters are expressed as a function of the axial strain *ε*.

**Figure 6:**
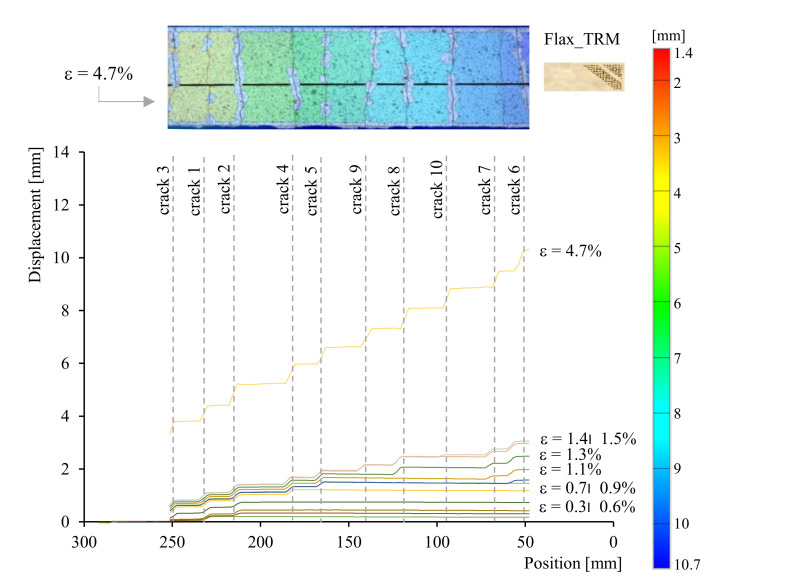
Displacement field of middle longitudinal section of a representative Flax TRM specimen.

Digital Image Correlation (DIC) analysis allows monitoring both the variables. Figure 6, obtained by DIC analysis, shows the axial displacement field of the middle longitudinal section at selected values of the strain, for a representative Flax TRM specimen. In addition, the top of the image shows the displacement field all over the external surface of the specimen corresponding to the strain at the failure *ε_max_*. The discontinuities in the displacement correspond to the crack location, while their magnitude is the crack’s width.

This representation provides: the number of cracks, their position along the specimen length, thus the distance between consecutives cracks, the triggering instant of the crack (with the corresponding strain, *ε*), the crack width and its evolution during the test. DIC analysis, being particularly time-consuming, is carried out for few representative specimens. To analyse the crack development pattern for all the tested specimens, a simplified analytical model for the crack width identification is adopted. This model assumes that the entire tensile strain of the composite corresponding to stages II and III is concentrated in the cracked sections, and that all the cracks have the same width. Consequently, the mean crack width, *w_m_(ε)*, expressed as a function of the axial strain, *ε*, takes the form shown in Equation (1):

(1) $$w_{m}\left(\varepsilon \right)=\frac{\left(\varepsilon -\varepsilon_{1}\right)\cdot L}{n{{}^{\circ}}_{cracks}\left(\varepsilon \right)}with\;\varepsilon_{1}\leq \varepsilon \leq \varepsilon_{\max}$$

where *ε_1_* is the axial strain at the transition point between stages I and II, *L* is the gauge length equal to 300 mm and *n°_cracks_(ε)* is the number of cracks along the specimen at a given strain *ε*, assessed from the stress-strain curves considering that a crack occurrence corresponds to each peak in stage II.

**Figure 7:**
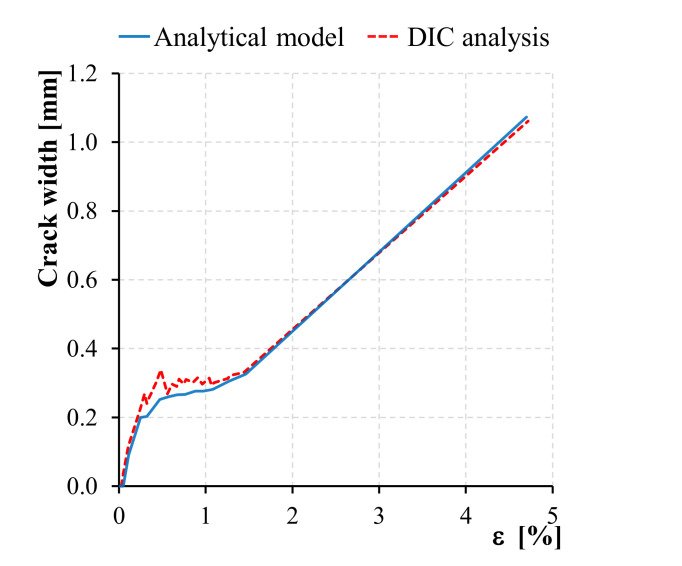
Crack width assessment: comparison between DIC analysis and analytical model.

Values of mean crack width of a representative Flax TRM specimen can be either obtained from DIC measurement or the analytical formula in Equation (1): Figure 7 shows a comparison between the values obtained by means of these two alternative methods. It is clear that in this specific case, due to the relatively lower stiffness of the textile with respect to the matrix, the simplified model accurately reproduces the mean crack width evolution during the test.

Although a similar study proved the efficiency of the proposed simplified method [43], further analysis is needed to validate it with respect to larger populations of specimens, and with different composite systems.

**Figure 8:**
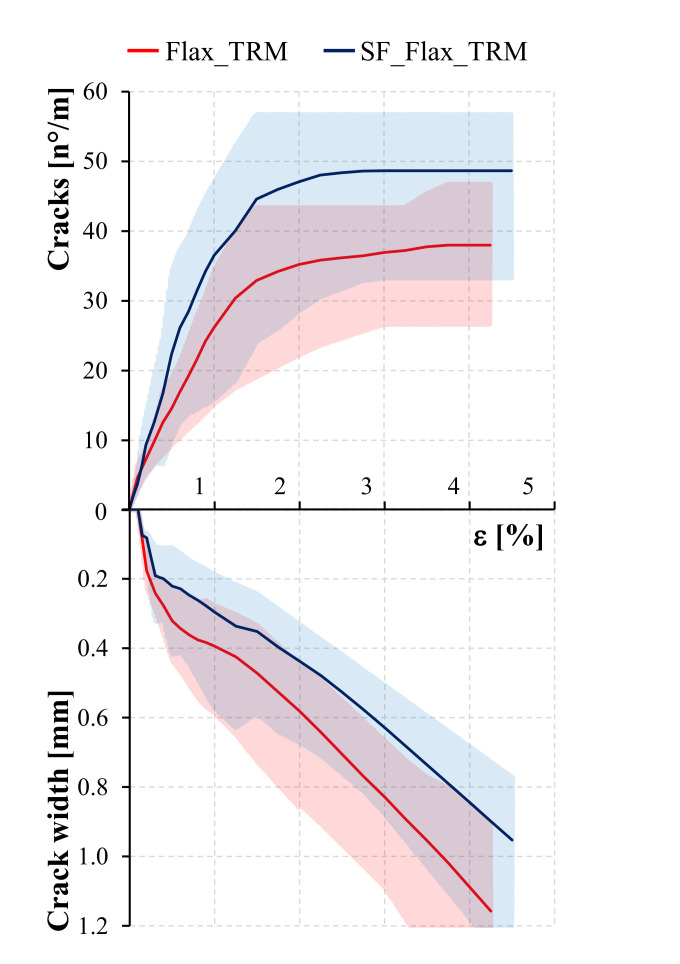
Mean number of cracks and crack width for Flax TRM and SF_Flax_TRM series.

The top of Figure 8 represents the evolution of the mean number of cracks for unit length as a function of the axial strain, *ε*, for both series of specimens. 

The graph in Figure 8 emphasises that, with the axial strain being equal, SF_Flax TRM specimens include a higher number of cracks. This evidence was not expected. In fact, literature studies show that TRM systems with equal textile reinforcement and a more resistant mortar tend to present more spaced cracks [44]. However, this discrepancy is due to the fact that in conventional TRMs, in proximity of a cracked section, the load is assumed to be entirely borne by the textile, and the contribution of the matrix is null. Conversely, in SF_Flax TRM specimens, instead, short curauá fibres create, in proximity of cracked sections, a bridging effect that makes the mortar contribute to the load bearing.

**Figure 9:**
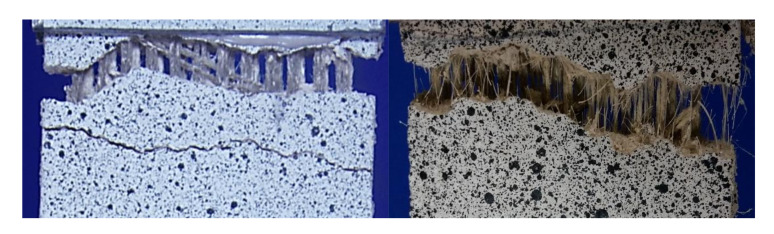
Close-up of a crack of Flax TRM and SF_Flax TRM representative specimens.

In Figure 9, showing the close-up of a crack of representative Flax TRM and SF_Flax TRM specimens, the curauá fibres’ bridging effect is emphasised.

The beneficial effect of the short fibre addition within the mortar is also shown in terms of crack openings. The bottom of Figure 8 shows the evolution of the mean crack width, as a function of the axial strain, *ε*, for both series of specimens. With the strain being equal, the SF_Flax TRM curve presents crack openings of lower magnitude during the whole test.

In fact, considering representative values of the strain equal to 1% and 2%, respectively corresponding to a generic point of stage II and the transition point between stages II and III, SF_Flax TRM specimens exhibit a mean value of the crack width about 25% lower than that concerning the reference series. The mean crack width corresponding to a strain of 4%, considered as representative of the failure of the specimens, assumes values of 1.1 and 0.8 mm respectively, for Flax TRM and SF_Flax TRM series of specimens, meaning that the addition of the short curauá fibres brings a reduction of the crack opening of about 22%. This evidence represents a significant aspect in view of application of the composite on a structural scale, where serviceability limit state and durability conditions may require restrictions in terms of maximum crack openings.

## 4. Conclusions

This paper showed the results of an experimental analysis aiming at assessing the influence of the use of short curauá fibres in the matrix of Flax TRMs on their tensile mechanical behaviour. The main outcomes of the study are listed as follows:

- The addition of short curauá fibre in the amount of 1% in weight increased the flexural strength of the mortar, but significantly reduced its workability at the fresh state: the addition of superplasticizer admixtures is needed to guarantee a proper implementation of the composite.
- Flax TRMs, either including or not including short curauá fibres, showed the typical tri-phase tensile behaviour of plant-based TRMs; moreover, the use of impregnated textile reduced the deformability of the composite in stage II, making its response closer to that of conventional TRMs with respect to plant-based TRMs, including non-treated textiles [15].
- SF_Flax TRM specimens, including the fibre-reinforced mortar, exhibited a higher value of the stress corresponding to the occurrence of the first crack, σ_1_, and in general, a higher average stress in the entire stage II. SF_Flax TRM specimens exhibited a mean value of the dissipated energy in stage II, *Γ_II_*, of about 45% higher than that shown in Flax TRM specimens.
- No specific influence of the short fibre addition was observed in terms of deformability of the composite (*ε_2_* and *ε_max_*): this aspect is mainly affected by the type of reinforcing textile adopted.
- SF_Flax TRM specimens exhibited a denser crack pattern with respect to Flax TRM specimens; in fact, due to the bridging effect of short curauá fibres, a general beneficial influence was observed, resulting in a higher number of cracks, and in a significant reduction of their width.

All in all, the study confirms Flax TRM as a valuable sustainable composite system for structural strengthening. Moreover, the study highlighted that the use of fibre-reinforced mortar in such composite systems leads to a general improvement in their mechanical behaviour. Improved composites show a denser crack pattern with less significant drops of the load corresponding to a crack occurrence and, hence, a mechanical response closer to the ideal tri-linear behaviour of strain-hardening textile-reinforced composites. In addition, the use of short fibres made of curauá resulted in a very successful solution combining sustainable principles and mechanical performance.

The promising results of the study pave the way for further analysis aimed at confirming the efficiency of the proposed improvement solutions of the composite, also focusing on their influence toward durability aspects.

## Figures and Tables

**Table 1:** Mean values of physical and mechanical properties of fibres and mortars adopted.

	Properties	Units	Mean	Co.V. (%)
Flax thread				
	filament diameter	(mm)	16.8	30
	density	(g/cm^3^)	1.19	3
	linear density	(Tex)	302	15
	No. threads/cm	(threads/cm)	4.3	-
	cross section	(mm^2^)	0.25	17
	Young modulus	(GPa)	9.4	11
	strain to failure	(%)	3.9	13
	tensile strength	(MPa)	353.7	12
Impregnated flax textile strip				
	No. of threads per strip	(threads/strip)	12	-
	cross section	(mm^2^)	6.1	17
	Young modulus	(GPa)	12.4	14
	strain to failure	(%)	3.1	18
	tensile strength	(MPa)	266	13
Curauá fibres filament				
	diameter	(μm)	77.7	11
	length	(mm)	20	-
	tensile strength	(MPa)	697.2	22
Plain mortar				
	spreading	(mm)	223	2
	compressive strength	(MPa)	8.4	8
	flexural strength	(MPa)	3.9	8
Fibre-reinforced mortar				
	spreading	(mm)	153	2
	compressive strength	(MPa)	9.9	4
	flexural strength	(MPa)	5.9	4

**Table 2:** Mean values of: stress *σ_1_*, dissipated energy in stage II, Γ_II_, strains *ε_2_* and *ε_max_*.

Series	σ_1_ (MPa)	*ε_2_* (%)	*ε_max_* (%)	Γ_II_ (J)
Flax TRM	0.65	2.0	5.1	3.55
Co.V.	42%	40%	13%	24%
SF_Flax TRM	0.83	2.1	5.0	5.24
Co.V.	15%	26%	6%	12%

## Data Availability

The data presented in this study are available on request from the corresponding author.
